# Translation of Hepatitis A Virus IRES Is Upregulated by a Hepatic Cell-Specific Factor

**DOI:** 10.3389/fgene.2018.00307

**Published:** 2018-08-10

**Authors:** Akitoshi Sadahiro, Akira Fukao, Mio Kosaka, Yoshinori Funakami, Naoki Takizawa, Osamu Takeuchi, Kent E. Duncan, Toshinobu Fujiwara

**Affiliations:** ^1^Laboratory of Infection and Prevention, Department of Virus Research, Institute for Frontier Life and Medical Sciences, Kyoto University, Kyoto, Japan; ^2^Laboratory of Biochemistry, Graduate School of Pharmaceutical Sciences, Kindai University, Osaka, Japan; ^3^Laboratory of Virology, Institute of Microbial Chemistry (BIKAKEN), Tokyo, Japan; ^4^Center for Molecular Neurobiology, University Medical Center Hamburg-Eppendorf, Hamburg, Germany

**Keywords:** Hepatitis A Virus (HAV), internal ribosome entry site (IRES), translation regulation, tropism, translation initiation

## Abstract

Many viruses strongly prefer to infect certain cell types, a phenomenon known as “tropism.” Understanding tropism’s molecular basis is important for the design of vaccines and antiviral therapy. A common mechanism involves viral protein interactions with cell-specific surface receptors, but intracellular mechanisms involving translation have also been described. In this report, we focus on Hepatitis A Virus (HAV) tissue tropism from the standpoint of the translational machinery. HAV genomic RNA, like other positive stranded RNA viruses, is devoid of a cap structure and its translation is driven by highly structured RNA sequences termed internal ribosome entry site (IRES) in the 5′ untranslated region (UTR). Unlike most viral IRESs, HAV IRES-mediated translation requires eIF4E and the 3′ end of HAV RNA is polyadenylated. However, the molecular mechanism of HAV IRES-mediated translation initiation remains poorly understood. We analyzed HAV-IRES-mediated translation in a cell-free system derived from either non-hepatic cells (HeLa) or hepatoma cells (Huh-7) that enables investigation of the contribution of the cap and the poly(A) tail. This revealed that HAV IRES-mediated translation activity in hepatoma cell extracts is higher as compared to extracts derived from a non-hepatic line. Our data suggest that HAV IRES-mediated translation is upregulated by a hepatic cell-specific activator in a poly(A) tail-independent manner.

## Introduction

In eukaryotes, the vast majority of cellular mRNAs are capped at the 5′ end and polyadenylated at the 3′ end. Translation initiation on these mRNAs follows a well-defined pathway (Green et al., 2016) involving multiple stages governed by a large number of eIFs interacting with the mRNA and the ribosome (Jackson et al., 2010). The first step is recognition of the m^7^G cap structure by the eukaryotic initiation factor (eIF) 4F complex, which consists of an ATP-dependent RNA helicase eIF4A, a large scaffold protein eIF4G, and a cap binding protein eIF4E. eIF4G interacts with the poly(A) binding protein PABP and the interaction between these three proteins (eIF4E, eIF4G, and PABP) leads to circularization of the capped mRNA (Tarun and Sachs, 1995; Le et al., 1997; Imataka et al., 1998). The 43S pre-initiation complex (PIC), which consists of the ternary complex (eIF2–GTP–Met–tRNAi), several eIFs including eIF1, 1A, 3, and 5, and the 40S small ribosomal subunit, is recruited to the mRNA via the eIF4F complex.After binding to mRNA, the 43S PIC scans the mRNA 5′ UTR in a 5′ to 3′ direction until the Met–tRNAi recognizes the start AUG codon. The recognition of the start AUG codon leads to conformational changes that produce a stable 48S initiation complex. Subsequently some eIFs are released, and then the 60S large ribosome subunit is recruited to form an 80S initiation complex ready to synthesize encoded peptide.

Capping and polyadenylation of cellular mRNA occurs in the nucleus, co-transcriptionally (Moteki and Price, 2002; Richard and Manley, 2009). However, many RNA viruses replicate in the cytoplasm and viral proteins are synthesized from their mRNA without cap structure. Picornaviruses that are positive-stranded RNA viruses replicate in the cytoplasm, but their mRNAs have a poly(A) tail synthesized by a viral RNA polymerase. In contrast, picornavirus mRNA lacks a cap structure. Instead of cap structure, a viral protein (VPg) is covalently linked to the 5′ end. Picornavirus mRNA is translated via an internal ribosome entry site (IRES) in its 5′ UTR. Viral IRES are highly structured RNA sequences that recruit the ribosome onto mRNA in a cap independent manner. IRES elements were initially reported in poliovirus (PV) and encephalomyocarditis virus (EMCV) genomes (Jang et al., 1988; Pelletier and Sonenberg, 1988). It has been demonstrated that the viral mRNAs of all members of the *Picornaviridae* family are translated in an IRES dependent manner (Martinez-Salas et al., 2015). Picornavirus IRESs can be classified into three groups by eIFs/cellular proteins requirement and scanning mechanism. The picornavirus type I IRESs (e.g., Enterovirus, Rhinovirus) and type II IRESs (e.g., Cardiovirus and Aphthovirus) require almost all of initiation factors, except for eIF4E, to stimulate the IRES activity. Moreover, some cellular proteins, known as IRES trans-acting factors (ITAFs) stimulate the type I and II IRESs activity. ITAFs have typically been identified using biochemical approaches. They are usually RNA-binding proteins (e.g., polypyrimidine tract binding protein; PTB) and can regulate IRES-dependent translation both positively and negatively (Graff et al., 1998; Hunt and Jackson, 1999; Gosert et al., 2000; Yi et al., 2000; Cordes et al., 2008). The picornavirus type I and type II IRES are classified by scanning mechanism. The 3′ border of type I and II IRES harbors the Yn-Xm-AUG motif in which Yn (pyrimidine-rich tract; *n* = 8–10 nt) is separated by a spacer (Xm; m = 18–20 nt spacer) from an AUG triplet. In the case of type I IRES, recruited ribosome to Yn-Xm-AUG motif scans the downstream spacer region (at a distance of ∼160 nt) for translation initiation on the authentic AUG (Hellen et al., 1994). On the other hand, in the case of type II IRES, ribosome is recruited to AUG codon of Yn-Xm-AUG motif directly without scanning (Kaminski et al., 1994). The picornavirus IRES type III (e.g., HAV) requires all of initiation factors including eIF4E and some ITAFs to stimulate the IRES activity.

The host and tissue tropisms of viruses are determined by multiple host and viral factors. In general it is well accepted that there are two major types of viral tropism, receptor-dependent and -independent tropism. The translation machinery is one of the important factors for determining receptor-independent tropism, since the host translation machinery is used to express viral proteins, and thus, to regulate viral propagation (Niepmann, 2009). Tissue-specific expression of ITAFs could help to explain viral tissue tropism on the translational level. For example, ITAF_45_, also known as erbB-3-binding protein 1 (Ebp1), binds to foot-and-mouth disease virus (FMDV) IRES and stimulates IRES activity. ITAF_45_ expresses in proliferating cells during the S phase but not during cell cycle arrest (Radomski and Jost, 1995). Therefore, FMDV IRES is activated only in proliferating tissue, characterizing the virus as endotheliotropic (Pilipenko et al., 2000). miR-122 that is expressed preferentially in liver cells is one of the *trans*-acting factors for regulating HCV translation (Lagos-Quintana et al., 2002; Sempere et al., 2004; Chang et al., 2004; Fu et al., 2005). miR-122 contributes to stimulation of HCV translation by recognition of a *cis*-acting element on the 5′ UTR of its mRNA, and thus, miR-122 is one factor determining HCV liver tropism on the level of translation (Henke et al., 2008).

Hepatitis A Virus (HAV), a member of the *Picornaviridae* family, is one of the major causative agents of acute hepatitis. HAV infection does not cause chronic liver disease, but superinfection of HAV with hepatitis B virus or HCV may affect the natural history of HBV and HCV related to liver cirrhosis and cancer (Lemon et al., 2017). HAV mRNA contains an IRES element in its 5′ UTR, a single open reading frame, and is polyadenlyated (Brown et al., 1991). The translational mechanism driven from Picornaviral IRES is determined by characteristic of the IRES element. IRES elements of picornaviruses are categorized into three groups and the HAV IRES belongs to type III. In the case of HAV IRES-mediated translation, the first event of translation initiation is that eIF4F complex recognizes the IRES element (Borman et al., 2001). Interestingly, it has been demonstrated that eIF4E, the cap binding protein, is required for activation of HAV IRES-mediated translation although HAV mRNA does not have cap structure (Ali et al., 2001). Subsequently the 43S PIC and the 60S large ribosome subunit are recruited onto HAV mRNA, followed by synthesis of the viral polyprotein. HAV IRES activity was found to be stimulated by two ITAFs, PTB and poly(rC) binding protein 2 (PCBP2) (Graff et al., 1998; Gosert et al., 2000). Conversely, two negative ITAFs that repress HAV IRES activity were identified: GAPDH and La autoantigen (Yi et al., 2000; Cordes et al., 2008). It was suggested that HAV IRES-mediated translation could explain hepatotropism (Borman et al., 1997). However, the reported positive and negative ITAFs for HAV translation are broadly expressed in many tissues. Thus, the hepatocyte specific translational mechanism of HAV mRNA remains unclear.

In this study, we focus on hepatic cell-specific translation mediated by the HAV IRES. We previously established a cell-free translation system using human cervical carcinoma cell (HeLa) extracts (Fukao et al., 2009). Thus, to analyze hepatic cell-specific translation mechanisms, we adapted this system to the human hepatoma cell (Huh-7) and established a cell-free translation system using Huh-7. By measuring HAV IRES-mediated translation activity in Huh-7 and HeLa cell extracts, we show that an HAV IRES reporter mRNA is translated more efficiently in Huh-7 cell extracts than in HeLa cells. Translation extract mixing experiments demonstrate that this effect is due to a positive-acting factor in the Huh-7 cell extracts. Moreover, translational enhancement mediated by the HAV IRES in Huh-7 cell extracts was also observed with a reporter mRNA lacking a poly(A) tail. Our results support the idea that HAV is highly hepatotropic on a translational level and suggest that liver cell-specific components can stimulate translation of this IRES through a mechanism that does not involve the poly(A) tail.

## Materials and Methods

### Plasmids

Reporter plasmid (pBSII-Nluc-A114) encoding NanoLuc (Nluc) luciferase was constructed previously (Fukao et al., 2014). To obtain the plasmid encoding HAV-IRES-Nluc-A114 and EMCV IRES-Nluc-A114 (pBSII-HAV-IRES-Nluc-A114 and pBSII-EMCV-IRES-Nluc-A114), the total synthesized HAV-IRES sequence (Borman et al., 1995) (invitrogen) and the total synthesized EMCV-IRES were inserted upstream of Nluc gene of pBSII-Nluc-A114.

### *In vitro* Transcription

Plasmids were linearized downstream of A114 or the Nluc gene with HindIII or XbaI, respectively. Linearized plasmids with HindIII or XbaI were used as templates to synthesize mRNA with or without a poly(A) tail. mRNAs were synthesized in the presence of either 7mGpppG (cap), ApppG (Acap) or in the absence of cap analog (Nocap) with previously described protocol(Duncan et al., 2009). The synthesized RNAs were purified by RNeasy Mini Kit (QIAGEN).

### Cell Culture and *in vitro* Translation

Huh-7 and HeLa cells were cultured in Dulbecco’s modified Eagle’s medium (Gibco) supplemented with 5% fetal bovine serum. Cells were detached for preparation of cell extracts by 2.5 g/l-Trypsin Solution (Nacalai) when cell outgrowth had reached 90% confluence. Detached cells were collected by centrifugation at 700 × *g* for 2 min and washed two times in phosphate buffered saline (PBS) at 4°C. Pelleted cells were suspended in 1 volume of ice cold lysis buffer (Thoma et al., 2004). After 5 min on ice, cells were lysed with 12 strokes with needle. Following centrifugation of the homogenate at 10,000 × *g* for 10 min at 4°C, supernatant was collected as cell extracts. These cell extracts were dialyzed with lysis buffer, and dialyzed cell extracts were used for translation reaction. A total of 8 μl of nuclease-untreated HeLa or Huh-7 cell extracts, 10 μl of reaction buffer (30 mM Hepes-KOH buffer [pH 7.4], 8 mM creatine phosphate, 0.5 mM spermidine, 1 mM ATP, 0.2 mM GTP, 20 μM amino acids, 1.5 mM magnesium acetate, 80 mM potassium acetate, 40 μg/ml creatine kinase), and 2 μl of 10 ng/μl reporter mRNA were incubated at 37°C for 30 min. The reaction was stopped by liquid N_2_, and the luciferase reporter assay was performed according to the manufacturer’s protocol (NanoGlo Luciferase Assay Systems, Promega).

### Northern Blot Analysis

Total RNA was extracted from *in vitro* translation reaction mixtures after luciferase re- porter assays using ISOGEN II (Nippon Gene). Samples were separated in a 1.0% formaldehyde-containing agarose gel and transferred onto a nylon membrane (Pall). After blotting RNAs onto nylon membrane, the membrane was dyed by methylene blue solution (0.04% methylene blue, 0.5M NaOAc [pH 5.3]). The stained membrane was subjected to analysis under bright field with the LAS-4000 image analyzer (Fuji). Reporter mRNAs were detected with digoxigenin (DIG)-labeled RNA probe complementary to the Nluc gene in hybridization buffer (50% formamide, 750 mM NaCl, 75 mM Trisodium Citrate, 2% Blocking Reagent, 0.1% SDS, 0.1% *N*-Lauroylsarcosine, 200 ng/μl yeast tRNA) at 60°C for 12 h. The hybridization signal was detected with CDP Star (Roche) as the reaction substrate according to the manufacturer’s instructions. The membrane was subjected to analysis with an LAS-4000 image analyzer.

### Sucrose Density Gradient Assay

*In vitro* translation was scaled up to 40 μl and performed at 37°C for 10 min. After 10 min incubation, 0.5 μl of 100 mg/ml cycloheximide was added to stop the translation reaction. The reaction mixture was loaded on the top of 11 ml of a linear 5–25% sucrose gradient (5–25% sucrose in 20 mM HEPES-KOH [pH 7.6], 150 mM potassium acetate, 5 mM MgCl_2_). After centrifugation at 38,000 rpm for 2.5 h at 4°C in a HITACHI P40ST rotor, 11 fractions (each 1 ml) were collected from the top of the gradient using a piston gradient fractionator (BIOCOMP). Total RNA was extracted from each fraction, and Nluc mRNA was detected by Northern blotting.

## Results

### Cap- and Poly(A)-Dependent Translation Huh-7 Cell Extracts

**FIGURE 1:**
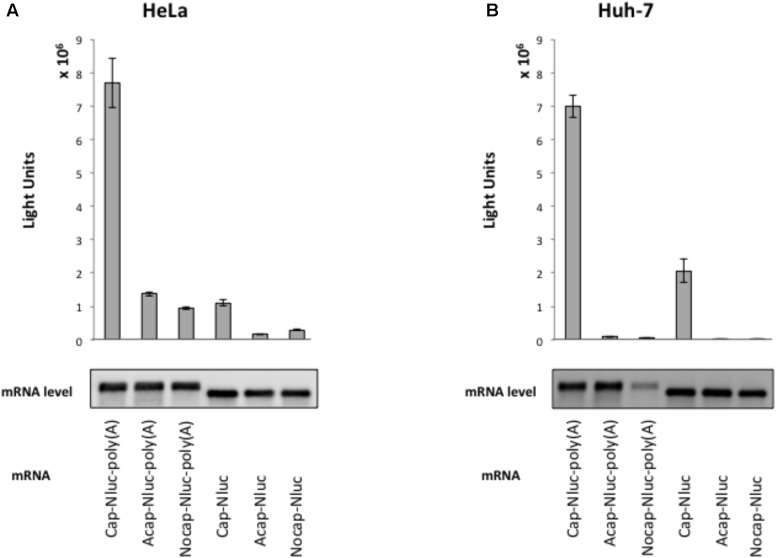
Cap structure and poly(A) tail synergy in the HeLa- and Huh-7-derived cell free translation system. (top panel) HeLa **(A)** or Huh-7 **(B)** translation extracts were mixed with the indicated Nanoluc (Nluc) reporter mRNAs at 37°C for 30 min. The luciferase light units are shown. Error bars reflect the standard deviation of values in at least three independent experiments. (bottom panel) Physical stabilities of the indicated mRNAs at the end of the incubation were analyzed by Northern blotting.

To elucidate HAV IRES-mediated translation preference for cell extracts, we established a cell-free translation system from HeLa cell and human liver cell (hepatoma) Huh-7 extracts. To assess our *in vitro* translation system, we measured Nluc activity from reporter mRNAs with an m^7^GpppG cap, analogous to the physiological cap, or a non-physiological ApppG cap analog (Acap) that is not recognized by eIF4E and with or without poly (A) tail. Reporter mRNAs were incubated with HeLa and Huh-7 cell extracts at 37°C for 30 min. In HeLa cell extracts, translation activity of cap-Nluc-poly(A) mRNA was approximately 7 times higher than that of cap-Nluc mRNA, and 5.5 times higher than that of Acap-Nluc-poly(A) mRNA (**Figure 1A**). In Huh-7 cell extracts, translation activity of cap-Nluc-poly(A) mRNA was approximately 3.5 times higher than that of cap-Nluc mRNA, and 75 times higher than that of Acap-Nluc-poly(A) mRNA (**Figure 1B**). Moreover, translation activity of the other reporter mRNAs [Nocap-Nluc-poly(A), Acap-Nluc, Nocap-Nluc] were 8–500 times lower than cap-Nluc-poly(A) mRNA. In principle, differences in mRNA stability could contribute to the observed differences in luciferase activity. To address this issue, we measured reporter mRNA levels at the end of the different translation reactions. We detected no significant differences in the amount of reporter mRNAs in HeLa (**Figure 1A**). On the other hand, in Huh-7 cell extracts, Nocap-Nluc-poly(A) mRNA was degraded after translation reaction, but the other reporter mRNAs were stable after translation reaction (**Figure 1B**). These data indicates that the observed differences of translation activity are due to different translational efficiencies, except for Nocap-Nluc-poly(A) in Huh-7. These results show that our *in vitro* translational system with HeLa and Huh-7 extracts can analyze cap- and poly(A)-dependent translation.

### HAV IRES-Mediated Translation Is Enhanced in Huh-7 Cell Extracts

**FIGURE 2:**
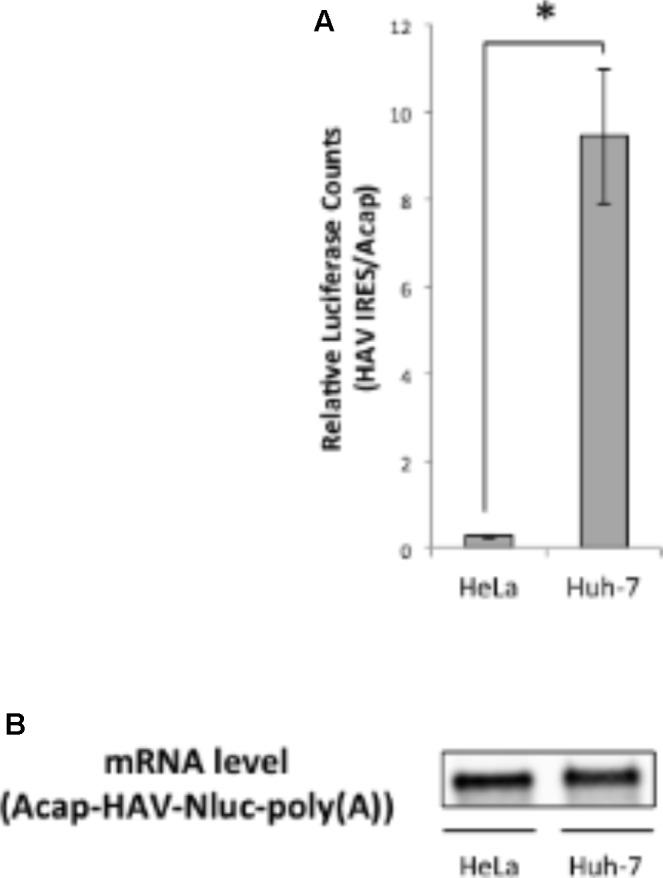
Translation driven from HAV IRES in Huh-7 *in vitro* translation system is more efficient than in HeLa. **(A)** HeLa or Huh-7 translation extracts were mixed with Acap-HAV-IRES-Nluc-poly(A) or Acap-Nluc-poly(A) reporter mRNAs. HAV IRES-mediated translation was normalized by Acap-Nluc-poly(A) mRNA translation. The mean values ±SD from three independent experiments are shown. The asterisk indicates a statistically significant difference (*p* < 0.05). **(B)** Physical stabilities of the Acap-HAV-IRES-Nluc-poly(A) mRNA at the end of the incubation were analyzed by Northern blotting.

Next, we analyzed whether translation mediated by the HAV IRES is enhanced in liver-derived hepatoma cells by comparing *in vitro* translation activities of HAV IRES reporters in HeLa or Huh-7 extracts. We used a monocistronic Nluc reporter mRNA containing a HAV IRES element in the 5′ UTR, as this configuration has been argued to be superior to dicistronic mRNAs for evaluating IRES-mediated translation (Song et al., 2006; Jünemann et al., 2007). The HAV IRES reporter mRNA not attached cap analog was unstable after 30 min incubation in our Huh-7 *in vitro* translation system, compared with a non-canonical Acap attached reporter mRNA (**Supplementary Figure S1**). Therefore, to analyze HAV IRES mediated translation and to inhibit reporter mRNA degradation, an Acap is attached to the 5′ end of the reporter mRNA which conjugated HAV IRES element. The translation activity of Acap-HAV-IRES-Nluc-poly(A) mRNA in Huh-7 cell extracts was approximately 35 times higher than in that in HeLa cell extracts (**Figure 2A**). Moreover, the translation activity driven by HAV IRES was analyzed in another liver-derived hepatoma cells, HepG2. Compared with HeLa cell, HAV IRES-mediated translation was enhanced in HepG2 cell extracts (**Supplementary Figure S2**). To determine whether this was due to effects on mRNA stability or translation, we again examined the amount of reporter mRNA after translation reactions by Northern blotting. The indicated reporter mRNA [Acap-HAV-Nluc-poly(A)] level in Huh-7 cell extracts was slightly lower than in HeLa cell extracts (**Figure 2B**). On the other hand, the reporter mRNA level in HepG2 cell is similar to those in HeLa cell extracts (**Supplementary Figure S3**). These results imply that HAV IRES-mediated translation is more efficient in extracts from the liver-derived Huh-7 or HepG2 cells than in HeLa, which have a cervical origin, not because of mRNA stability but because of translation regulation.

### 80S Ribosome Assembly on a HAV IRES Reporter mRNA Is More Efficient in Huh-7 Extracts

**FIGURE 3:**
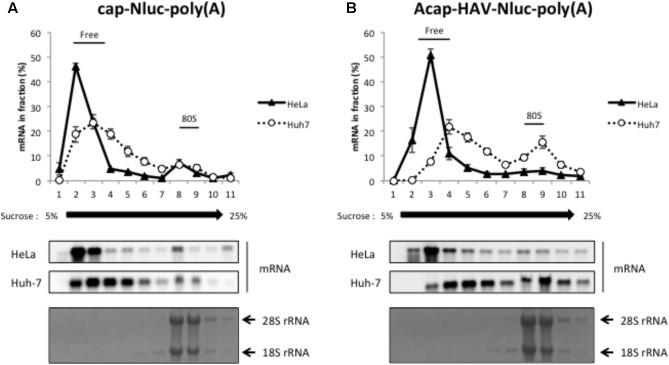
HAV IRES-mediated translation is stimulated in Huh-7 cell extracts at the level of 80S initiation complex formation. Sucrose density gradient assay of initiation complexes formed after 10 min incubation in HeLa (black triangle) or Huh-7 (open circle) cell extracts with the cap-Nluc-poly(A) reporter mRNA **(A)** or Acap-HAV-IRES-Nluc-poly(A) reporter mRNA **(B)**. (top panel) The amount of reporter mRNA in each fraction is indicated as a percent of total mRNA signal. The mean values ±SD from three independent experiments are shown. (middle two panels) The reporter mRNAs of each fraction were detected by Northern blotting using Nluc probe. (bottom panel) rRNAs were detected by methylene blue stain.

We next performed sucrose density gradient assays to analyze which step of HAV IRES-mediated translation might be more efficient in Huh-7 cell extracts vs. HeLa cell extracts. 28S rRNA and 18S rRNA, the components of 60S and 40S ribosomal subunits respectively, were mainly collected at fraction number 8-9 (bottom panels of **Figures 3A,B**). Thus, we defined fraction number 8–9 as the 80S fraction. Cap-Nluc-poly(A) mRNAs were mainly present in the ribosome-free fractions (fraction number 2–4) in both HeLa and Huh-7 cell extracts (**Figure 3A**). However, approximately 10% of cap-Nluc-poly(A) mRNAs were present with 80S fractions (fraction number 8–9) in HeLa and Huh-7 cell extract, indicating that the 80S initiation complex formed on the added mRNA suggesting that the mRNAs were translating. When Acap-HAV-IRES-Nluc-poly(A) mRNAs were used as reporters, only a small amount of Acap-HAV-IRES-Nluc-poly(A) mRNAs were present in 80S fractions in HeLa cell extracts, while approximately 20% of the reporter mRNAs were present in 80S fractions in Huh-7 cell extracts (**Figure 3B**). These results suggest that the 80S initiation complex is formed much more efficiently on the Acap-HAV-IRES-Nluc-poly(A) mRNA in Huh-7 extracts than in HeLa extracts. Thus, the difference in translational efficiencies between these two extracts is mediated mostly or exclusively at the level of translation initiation.

### A HAV IRES Reporter mRNA Lacking a Poly(A) Tail Is Still Translated More Efficiently in Huh-7 Cell Extracts Than in HeLa Cell Extracts

**FIGURE 4:**
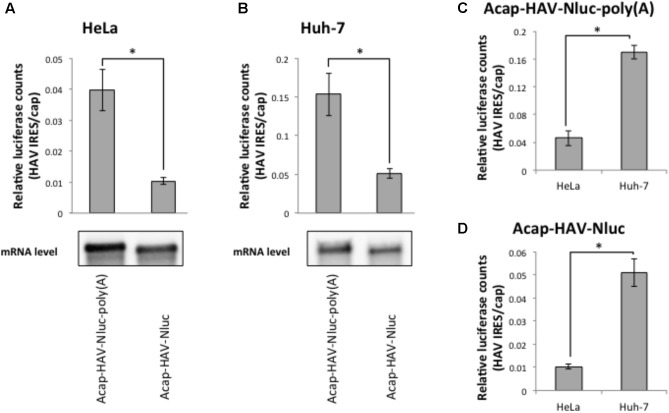
Enhanced activity of the HAV IRES in Huh-7 vs. HeLa extracts is independent of the poly(A) tail. (top panel) HeLa **(A)** or Huh-7 **(B)** *in vitro* translation were performed with Acap-HAV-IRES-Nluc-poly(A) or Acap-HAV-IRES-Nluc reporter mRNAs. HAV IRES-mediated translation was normalized by cap-dependent translation. The mean values ±SD from three independent experiments are shown. (bottom panel) Physical stabilities of the Acap-HAV-IRES-Nluc-poly(A) and Acap-HAV-IRES-Nluc mRNAs at the end of the incubation were analyzed by Northern blotting. **(C)** Comparison of Acap-HAV-IRES-Nluc-poly(A) relative light units between in HeLa and in Huh-7 cell extracts. **(D)** Comparison of Acap-HAV-IRES-Nluc relative light units between in HeLa and in Huh-7 cell extracts. The asterisks indicate in **(A–D)** statistically significant differences (*p* < 0.05).

The HAV mRNA is polyadenylated and the poly(A) tail is a known translational enhancer element. Thus, we next asked whether the enhanced HAV IRES-mediated translation in Huh-7 cell extracts depended on a poly(A) tail. We compared the translational activity of HAV IRES reporter mRNAs with or without poly(A) tails in HeLa and Huh-7 cell extracts. In both types of extracts, the relative translational activity of Acap-HAV-IRES-Nluc mRNA was 3–4 times lower than that of Acap-HAV-IRES-Nluc-poly(A) (**Figures 4A,B**). This was due to differences in translation efficiency, not mRNA stability, since levels of the reporter mRNAs at the end of the reactions were similar (**Figures 4A,B**). These results show that the poly(A) tail promotes translation of HAV IRES-containing mRNAs in both HeLa and Huh-7 cell extracts.

Next, we compared relative translational activity of the HAV IRES reporter mRNA lacking a poly(A) tail in HeLa cell extracts and Huh-7 cell extracts. The enhancement of translation mediated by HAV IRES in Huh-7 cell extracts still occurred even when the poly(A) tail was deleted from the reporter mRNAs (**Figures 4C,D**). This result clearly demonstrates that the difference in translational efficiencies of the HAV IRES in Huh-7 cell extracts vs. HeLa cell extracts is independent of the poly(A) tail.

### Huh-7 Cell Extracts Contain an Activator of HAV IRES-Mediated Translation

In principle, there are two different mechanistic explanations for the difference in HAV IRES translational efficiency in Huh-7 and HeLa extracts: (i) HAV IRES-mediated translation is repressed in HeLa cell extracts by a repressor or (ii) HAV IRES-mediated translation is stimulated in Huh-7 cell extracts by an activator. To distinguish between these possibilities, we performed *in vitro* translation assays where we mixed a constant amount of Huh-7 cell extracts with increasing amounts of HeLa cell extract and *vice versa*.

**FIGURE 5:**
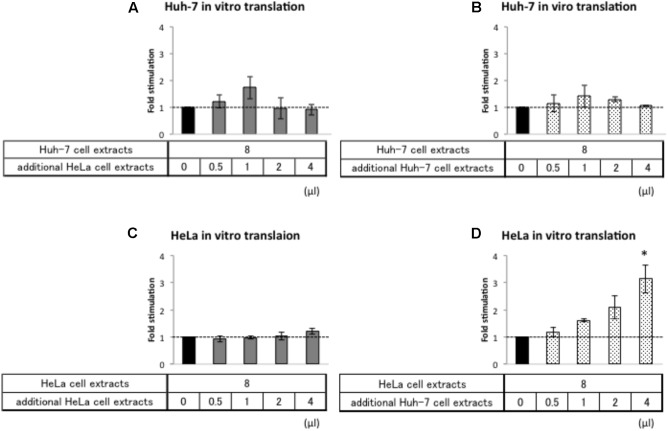
Huh-7 cell extracts contain a factor that promotes HAV IRES-mediated translation. Huh-7-based **(A,B)** or HeLa-based **(C,D)** *in vitro* translation reaction were performed with Acap-HAV-IRES-Nluc-poly(A) reporter mRNA. *In vitro* translation reactions were carried out in the presence of additional cell extracts (gray bars: HeLa, dotted bars: Huh-7) or absence of them (black bar). Each bottom panel indicates the volume of additional cell extracts added. The total volume of reaction mixture was not changed. HAV IRES-mediated translation was normalized by cap-dependent translation. Fold stimulation by additional cell extracts was calculated by dividing relative light units of Acap-HAV-IRES-Nluc-poly(A) obtained in translational effector-added translation reactions by those in lysis buffer-added translation reactions, which are set as 1 (symbolized as dotted line). The mean values ±SD from three independent experiments are shown. The asterisk indicates statistically significant differences in additional HeLa cell extracts **(C)** or Huh-7 cell extracts **(D)** in HeLa-based *in vitro* translation system (*p* < 0.05).

First, we analyzed whether addition of HeLa cell extracts repress relative Nluc activity from Acap-HAV-IRES-Nluc-poly(A) mRNA in the Huh-7 *in vitro* translation system. Addition of increasing amounts of HeLa cell extracts to Huh-7 extracts did not affect HAV IRES-mediated translation (**Figure 5A**). As a control, we also analyzed the effect of adding Huh-7 cell extracts in parallel (**Figure 5B**). This also did not have any additional effect, as expected. Thus, if HeLa cells do not contain a repressor, it is not active under these assay conditions. Next, we analyzed whether addition of Huh-7 cell extracts to HeLa cell extracts increased relative luciferase activity of Acap-HAV-IRES-Nluc-poly(A) mRNA. The relative luciferase activity from Acap-HAV-IRES-Nluc-poly(A) reporter mRNA was enhanced by addition of increasing amounts of Huh-7 cell extracts to HeLa cell extracts (**Figure 5D**). This was a specific effect of adding Huh-7 extracts, since addition of HeLa cell extracts to HeLa cell extracts did not affect the relative luciferase activity (**Figure 5C**). Taken together, these results strongly suggest that a component of Huh-7 cell extracts stimulates HAV IRES-mediated translation.

## Discussion

A fundamental aspect of viral biology is that different viruses infect different classes of cells, a phenomenon known as “tropism.” There is strong evidence that this can involve cell type-specific extracellular viral receptors, but there is also evidence that intracellular factors are involved (Henke et al., 2008; Schieck et al., 2013; Yan et al., 2013). Given the central importance of translation for viral replication, factors that regulate viral translation in a cell-selective manner could affect viral cell tropism. However, direct evidence for this idea has only been generated in a limited number of cases (Malnou et al., 2002; Guest et al., 2004). A limitation has been that most mechanistic studies using *in vitro* translation systems did not use extracts from the relevant cell types. In this study, we used cell-free translation systems to provide evidence for HAV hepatotropism at the translational level. By comparing HAV-IRES translational activity in extracts derived from target (liver) and non-target (cervical) cell types, we provide evidence for the existence of a factor in liver-derived cell types that functions in a poly(A) tail-independent manner to enhance translation initiation driven by the HAV IRES.

Hepatitis A Virus, a member of the picornavirus family, exhibits hepatotropism, meaning that it selectively infects liver cells, and thereby causes hepatitis A. Why does HAV infect liver cells selectively? Dotzauer et al. (2000) reported that liver-specific expression of the asialoglycoprotein receptor (ASGPR) contributes to hepatotropism of HAV infection through direct interaction with HAV-specific immunoglobulin A. However, for other viruses such as PV and HCV, IRES-mediated translation occurs in a tissue-specific manner driven by cell-specifically expressed *trans*-acting factors (Guest et al., 2004; Henke et al., 2008). Therefore, we hypothesized that HAV IRES-mediated translation might also be a mechanism underlying HAV hepatotropism. To investigate this hypothesis, we established an *in vitro* cell-free translation system from liver hepatoma Huh-7 cell extracts. We directly compared translational activity mediated by the HAV IRES in these extracts to parallel extracts derived from HeLa cells, a non-liver cell line. Previously, a cell-free translation system with rabbit reticulocyte lysates (RRL) mixed with HeLa cell extracts has been utilized to analyze the HAV IRES-mediated translation mechanism (Ali et al., 2001; Borman et al., 2001; Michel et al., 2001). We thought that a cell-free translational system from liver derived cells was more likely to capture natural regulation of the HAV IRES and thus would be useful to study hepatotropism at the translational level. Thus, we adapted a cell-free translation system involving only human cell extracts (Fukao et al., 2009) to Huh-7 cells. Both cap- and poly(A)-dependent translation occurred in our *in vitro* translation system (**Figure 1**). In this study, cell extracts were not treated with micrococcal nuclease because the HAV IRES-mediated translation activity was decreased by nuclease treatment (data not shown). The reason why nuclease treatment decreases HAV IRES-mediated translation in Huh-7 cell extracts is not known, but endogenous RNA, including mRNA and miRNA, may be required for HAV IRES-mediated translation.

It has been reported that translation mediated by HAV IRES is efficient in Huh-7 cells by evaluation of the translational level of HAV IRES-conjugated mRNA derived from transfected plasmids (Mackiewicz et al., 2010). Our data about HAV IRES-mediated translation provide direct evidence for this proposal. Moreover, the results of sucrose density gradient analysis emphasize that HAV IRES-mediated translation undergoes translation initiation more efficiently in Huh-7 cell extracts than in HeLa cell extracts. These data provide direct evidence that the HAV mRNA translational mechanism is more efficient in extracts from targeted cell types. On the other hand, EMCV mRNA translation was not enhanced in Huh-7 cell extracts, compared with HAV mRNA (**Supplementary Figure S3**). These data means that HAV IRES-mediated translation is enhanced specifically in targeted cell extracts. Translation enhancement is one of the contributing factors to viral tropism, expected to be partially determined HAV hepatotropism on the translational level.

All picornavirus mRNAs are polyadenylated, and the poly(A) tail of picornaviruses is necessary for infectivity and minus-strand RNA synthesis (Spector and Baltimore, 1974; Hruby and Roberts, 1976; Herold and Andino, 2001). In this study, we analyzed the poly(A) tail function for HAV IRES-mediated translation. Our data suggested that the poly(A) tail stimulates HAV IRES-mediated translation in HeLa cell extracts, consistent with a previous report (Bergamini et al., 2000). This result also indicates that our *in vitro* translation system can analyze the poly(A)-dependency of cap-independent translation. In Huh-7 cell extracts, we confirmed that the poly(A) tail promoted HAV IRES-mediated translation. These results suggest that HAV IRES-mediated translation can be stimulated by a poly(A) tail in Huh-7 cell as well as in HeLa cell extracts. The poly(A) tail interacts with PABP, which mediates efficient translation by circularization of HAV IRES mRNA via an IRES-eIF4G-PABP-poly(A) interaction (Michel et al., 2001). Our results show that HAV IRES mRNA without a poly(A) tail is nevertheless translated more efficiently in Huh-7 cell extracts than in HeLa cell extracts. Thus, efficient translation of HAV IRES mRNA in Huh-7 cell extracts does not depend on mRNA circularization via the poly(A) tail. Rather our data are most consistent with recognition of the HAV IRES element by a *trans*-acting factor in Huh-7 cells that stimulates translation in a poly(A) tail-independent manner.

Known IRES *trans*-acting factors (ITAFs) can either promote IRES-mediated translation or repress it (Graff et al., 1998; Gosert et al., 2000; Yi et al., 2000; Cordes et al., 2008). Moreover, for HCV, a specific miRNA is known to stimulate IRES-mediated translation (Henke et al., 2008). Our data clearly show that HAV IRES-mediated translation was promoted specifically by a liver cell-specific activator present in our Huh-7 cell translation extracts. This factor could be a liver-specific ITAF, miRNA, or a completely new class of translational regulatory factor. Identifying the factor will be crucial to fully elucidating the mechanism of HAV IRES-mediated translational enhancement in liver cells. We expect that our Huh-7 cell *in vitro* translation system will be instrumental for studying HAV mRNA translation. However, we also expect it to be useful for mechanistic analysis of liver-specific translational regulation of other mRNAs.

## Author Contributions

AS performed the measurements and he was involved in planning the work. AF performed the measurements and he was involved in planning and supervised the work. MK, NT, and YF aided in interpreting the results and worked on the manuscript. OT and KD processed the experimental data, performed the analysis, drafted the manuscript, and designed the figures. TF contributed to the design and implementation of the research, to the analysis of the results and to the writing of the manuscript. All authors discussed the results and commented on the manuscript.

## Conflict of Interest Statement

The authors declare that the research was conducted in the absence of any commercial or financial relationships that could be construed as a potential conflict of interest.
