# Supplementary figures and images for: Translation of Hepatitis A Virus IRES Is Upregulated by a Hepatic Cell-Specific Factor
Source: Front Genet. 2018 Aug 10;9:307. doi: 10.3389/fgene.2018.00307 (PMC6095998; doi:10.3389/fgene.2018.00307)

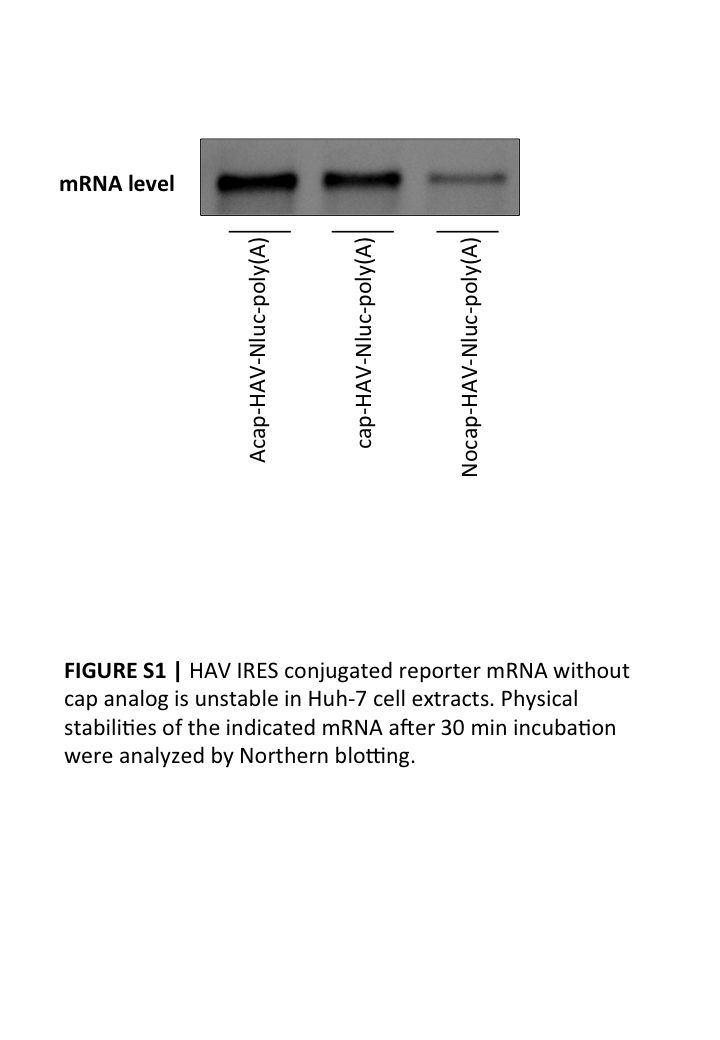

Supplement: Supplementary file 1 [file Image_1.tiff]

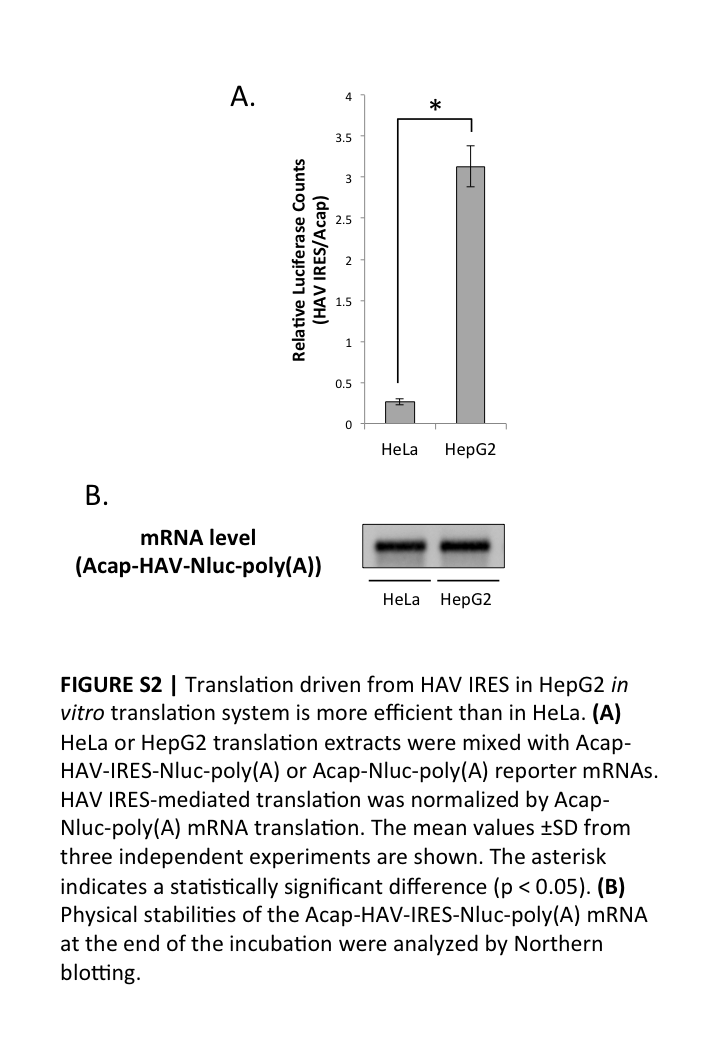

Supplement: Supplementary file 2 [file Image_2.tiff]

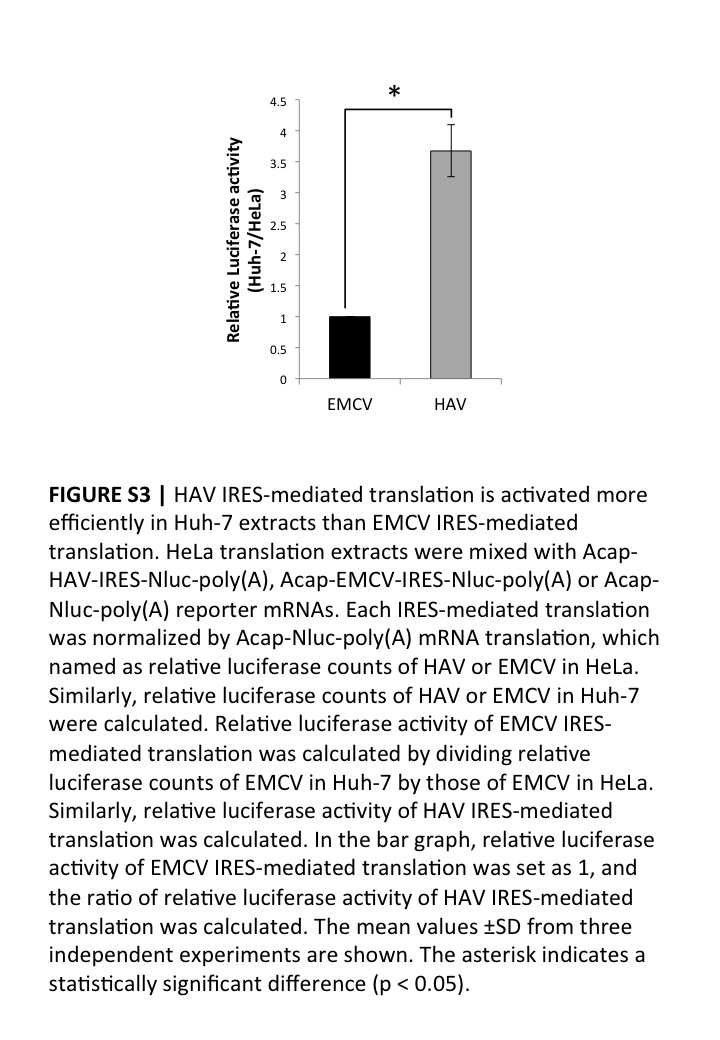

Supplement: Supplementary file 3 [file Image_3.tiff]
